# X-Ray Structure and Inhibition of 3C-like Protease from Porcine Epidemic Diarrhea Virus

**DOI:** 10.1038/srep25961

**Published:** 2016-05-13

**Authors:** Sarah E. St. John, Brandon J. Anson, Andrew D. Mesecar

**Affiliations:** 1 Department of Chemistry, Purdue University, West Lafayette, Indiana, USA; 2 Centers for Cancer Research & Drug Discovery, Purdue University, West Lafayette, Indiana, USA; 3 Department of Biological Sciences, Purdue University, West Lafayette, Indiana, USA

## Abstract

Porcine epidemic diarrhea virus (PEDV) is a coronavirus that infects pigs and can have mortality rates approaching 100% in piglets, causing serious economic impact. The 3C-like protease (3CL^pro^) is essential for the coronaviral life cycle and is an appealing target for the development of therapeutics. We report the expression, purification, crystallization and 2.10 Å X-ray structure of 3CL^pro^ from PEDV. Analysis of the PEDV 3CL^pro^ structure and comparison to other coronaviral 3CL^pro^’s from the same alpha-coronavirus phylogeny shows that the overall structures and active site architectures across 3CL^pro^’s are conserved, with the exception of a loop that comprises the protease S_2_ pocket. We found a known inhibitor of severe acute respiratory syndrome coronavirus (SARS-CoV) 3CL^pro^, (*R*)-**16**, to have inhibitor activity against PEDV 3CL^pro^, despite that SARS-3CL^pro^ and PEDV 3CL^pro^ share only 45.4% sequence identity. Structural comparison reveals that the majority of residues involved in (*R*)-**16** binding to SARS-3CL^pro^ are conserved in PEDV-3CL^pro^; however, the sequence variation and positional difference in the loop forming the S_2_ pocket may account for large observed difference in IC_50_ values. This work advances our understanding of the subtle, but important, differences in coronaviral 3CL^pro^ architecture and contributes to the broader structural knowledge of coronaviral 3CL^pro^’s.

The causative virus of porcine epidemic diarrhea in pigs was first described in England in 1978^1^, though the disease was first observed in 1971 among English feed pigs^2^. Subsequently termed porcine epidemic diarrhea virus (PEDV), it was classified as a coronavirus as a result of its appearance in electron microscopes and its polypeptide structure^3^,^4^,^5^. Before its occurrence in the United States, there were numerous reports of PEDV in various European countries, as well as in Korea, China, Japan, the Philippines and Thailand^6^. The first four reports of PEDV in the United States occurred within 10 days of each other on four farms in Iowa in late April of 2013^7^. The disease is characterized by porcine epidemic diarrhea with concomitant vomiting and dehydration, resulting in high fatality rates especially in piglets where mortality rates approach 100%^8^. The origin of transmission of PEDV to the United States is still unknown; however, in the past two years, it has resulted in a sizeable and negative impact on the US economy. The estimated net annual loss for the US economy as a result of PEDV, summed across all effects, is reported to range from $900 million to $1.8 billon as of June, 2014^9^.

Coronaviruses are enveloped, single-stranded, positive-sense zoonotic RNA viruses with genomes ranging from 27 to 32 kilobases in length, where the genes encoding for the structural proteins are located at the 3′ end and the replicase genes are located at the 5′ end of the genome. Two long ORFs encode for the replicase proteins, ORF1a and ORF1b, which are connected by a ribosomal frame-shift and produce the coronaviral polyproteins pp1a and pp1ab upon translation^10^. These viral polyproteins are processed at 14 cleavage sites by two or three cysteine proteases producing 16 non-structural proteins (nsps). The papain-like protease (PLP1 and PLP2 or PL^pro^) cleaves the polyprotein at 3 sites, whereas the 3C-like protease (3CL^pro^), also known as the main-protease (M^pro^) or nsp5, cleaves the polyprotein at 11 sites. The function of both proteases is essential for viral replication and therefore they are attractive targets for the development of anti-coronaviral therapeutics. In this study, we report the expression, purification, crystallization, and X-ray structure of PEDV-3CL^pro^ in its unbound state to 2.10 Å. We tested PEDV-3CL^pro^ for inhibition by (*R*)-**16**, a compound known to inhibit severe acute respiratory syndrome coronavirus (SARS-CoV) 3CL^pro^, and found (*R*)-**16** to inhibit PEDV-3CL^pro^ with an IC_50_ of 25.4 ± 1.4 μM. The structural information provided by the PEDV-3CL^pro^ X-ray crystal structure adds new details to our general understanding of the subtle differences between phylogenetically related 3CL^pro^’s and provides key insights that can be used to inform and guide the design of inhibitors against PEDV-3CL^pro^.

## Results and Discussion

**Figure 1:**
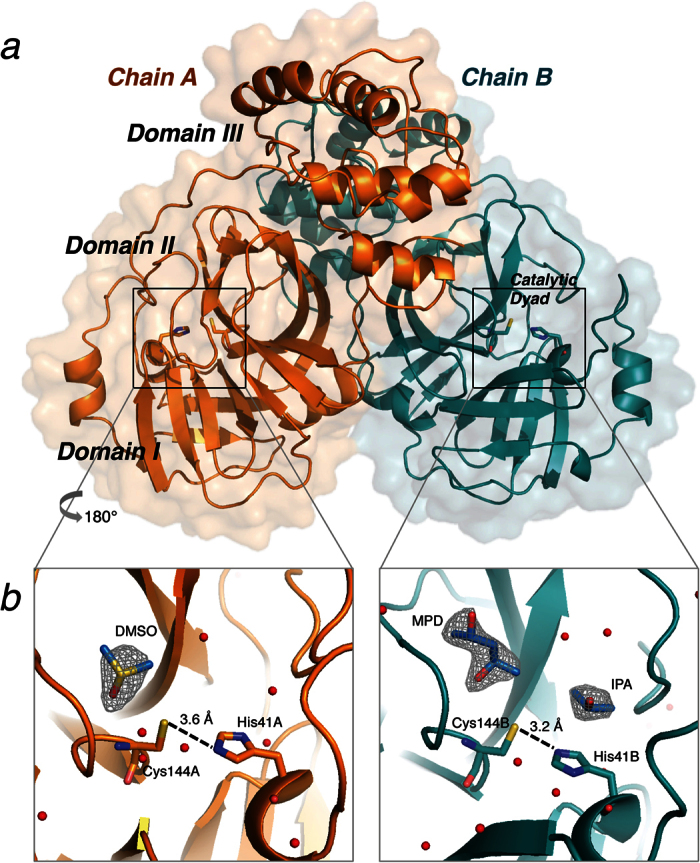
The X-ray crystal structure of PEDV-3CL^pro^ determined to 2.10 Å in its unbound state (PDB entry 5HYO). (**a**) The crystallographic dimer of PEDV-3CL^pro^ is shown with the backbone represented as a ribbon overlaid on a surface representation, where Chain A is colored in orange and Chain B is colored in deep slate. The active site catalytic dyad residues, His41 and Cys144, are represented as sticks in each monomer. The catalytic dyad is explicitly labeled in Chain B and domains I-III are labeled in Chain A. (**b**) Zoom-in of both Chain A and B active sites. The residues of the catalytic dyad are represented as sticks, colored by atom, and labeled accordingly. Waters are shown as non-bonded spheres and are colored red. Solvent molecules are shown as blue sticks, colored by atom, with surrounding electron density omit map (*F*_*o*_ − *F*_*c*_) shown in grey mesh and contoured to +3.0*σ* (DMSO = dimethyl sulfoxide, MPD = 2-methyl-2,4-pentanediol, IPA = isopropanol).

PEDV-3CL^pro^ crystallized in space group P2_1_2_1_2_1_ as a dimer in the asymmetric unit (Table 1). As reported for both human coronavirus 229E 3CL^pro^ and SARS-3CL^pro^, PEDV-3CL^pro^ is a homodimer containing three domains in each monomer (Fig. 1)^11^,^12^. The active site of PEDV-3CL^pro^, which contains a catalytic dyad formed from residues Cys144 and His41, is located in a cleft between domains I and II (Fig. 1a). Domain III is involved in monomer dimerization, which is ultimately responsible for forming the active protease (Fig. 1a)^12^,^13^. In the absence of substrate, water and solvent molecules (MPD, DMSO, and IPA) reside in the active site of PEDV-3CL^pro^, which is solvent exposed on one side (Fig. 1a,b). Interestingly, different solvent molecules are found in the respective active sites of the PEDV-3CL^pro^ monomers composing the PEDV-3CL^pro^ dimer, where the a single DMSO molecule resides in the Chain A active site and the Chain B active site houses both isopropanol (IPA) and 2-methyl-2,4-pentanediol (MPD) (Fig. 1b). The presence of different solvent molecules in each of the active sites of the dimer supports the assignment of one biological dimer in the asymmetric unit.

**Figure 2:**
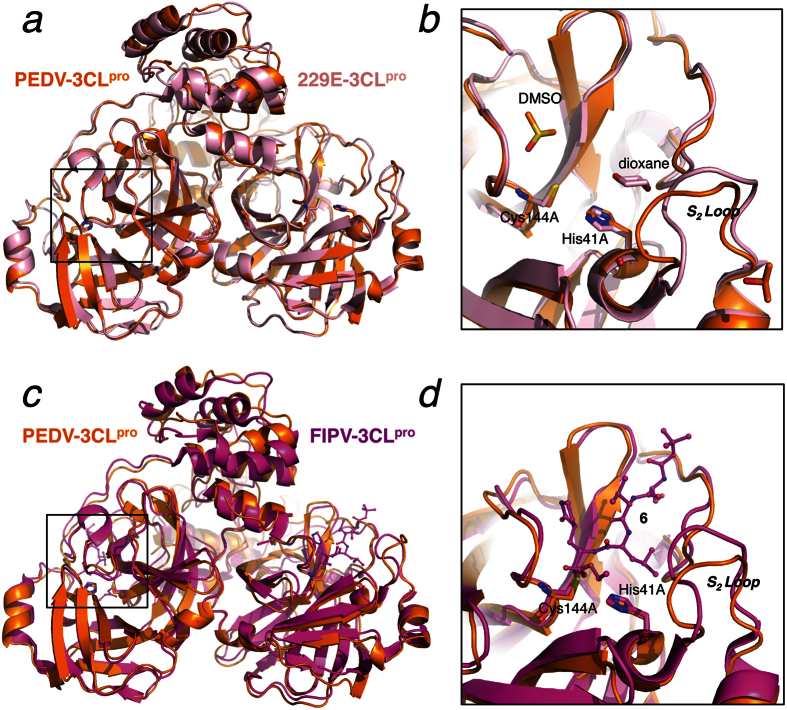
Superimpositions of PEDV-3CL^pro^, 229E-3CL^pro^ (PDB entry 1p9s), and FIPV-3CL^pro^ (PDB entry 4zro). (**a**) Superimposition of PEDV-3CL^pro^ and 229E-3CL^pro^, where each enzyme is represented as a ribbon. PEDV-3CL^pro^ is colored in orange and 229E-3CL^pro^ is colored light pink. (**b**) Zoom-in of PEDV-3CL^pro^:229E-3CL^pro^ superimposition Chain A active site, where catalytic dyad residues and solvent are shown in stick, colored according to atom and to which protein they belong. (**c**) Superimposition of PEDV-3CL^pro^ and FIPV-3CL^pro^, where each enzyme is represented as a ribbon. PEDV-3CL^pro^ is colored in orange and 229E-3CL^pro^ is colored magenta. (**d**) Zoom-in of PEDV-3CL^pro^:FIPV-3CL^pro^ superimposition Chain A active site, where catalytic dyad residues and solvent are shown in stick, colored according to atom and to which protein they belong. The covalent FIPV-3CL^pro^ inhibitor, **6**, is represented as ball-and-stick and colored according to atom.

Least-squares (LSQ) superposition of PEDV-3CL^pro^ and the unbound form of human coronavirus 229E 3CL^pro^ (PDB entry 1P9S)^12^, which are both from the same alpha-coronavirus phylogenetic group and share 69.3% sequence identity, resulted in an all-atom root-mean-square deviation (RMSD) value of 1.69 Å and a C-alpha RMSD value of 1.28 Å (Fig. 2a). The LSQ superposition of 229E-3CL^pro^ and PEDV-3CL^pro^ shows that the overall architectures of both the 229E-3CL^pro^ and PEDV-3CL^pro^ active sites in their unbound states are structurally very similar, and the active site residues of the catalytic dyad, residues Cys144 and His41 in both PEDV-3CL^pro^ and 229E-3CL^pro^, are located in almost identical structural space within the active site cavity, which is solvent exposed on one side (Fig. 2b). In the absence of substrate, both water and non-water solvent molecules (dioxane in 229E-3CL^pro^ and MPD, DMSO, and IPA in PEDV-3CL^pro^) are found in the active site (Fig. 2b).

In order to better understand the features of the PEDV-3CL^pro^ active site that are important in inhibitor and substrate binding, we generated an LSQ superposition of PEDV-3CL^pro^ and an inhibitor-bound form of feline infectious peritonitis virus 3CL^pro^ (FIPV-3CL^pro^, PDB entry 4ZRO), which belongs to the same alpha-coronavirus lineage as PEDV-3CL^pro^ and has 61.9% sequence identity (Fig. 2c)^14^. LSQ superposition resulted in an all-atom RMSD value of 2.11 Å and a C-alpha RMSD value of 1.69 Å. We found the overall active site architectures of the unbound PEDV-3CL^pro^ and the inhibitor-bound form of FIPV-3CL^pro^ to be remarkably similar with the catalytic dyad residues (Cys144 and His41 in both FIPV- and PEDV-3CL^pro^) in nearly identical orientations despite Cys144 of FIPV-3CL^pro^ being covalently modified by the inhibitor, compound **6** (Fig. 2d).

Interestingly, in both the superimpositions of PEDV-3CL^pro^ with 229E-3CL^pro^ and FIPV-3CL^pro^, the loops comprising the protease subsites of the 3CL^pro^ active site are in nearly identical structural locations, with the exception of the loop that comprises the S_2_ subsite, the S_2_ loop. The S_2_ loop forms the outer boundary of the S_2_ binding pocket and shows positional variability across the X-ray structures of 229E-, FIPV-, and PEDV-3CL^pro^, which may lead to differences in the size of the S_2_ subsites across 3CL^pro^’s (Fig. 2b,d).

**Figure 3:**
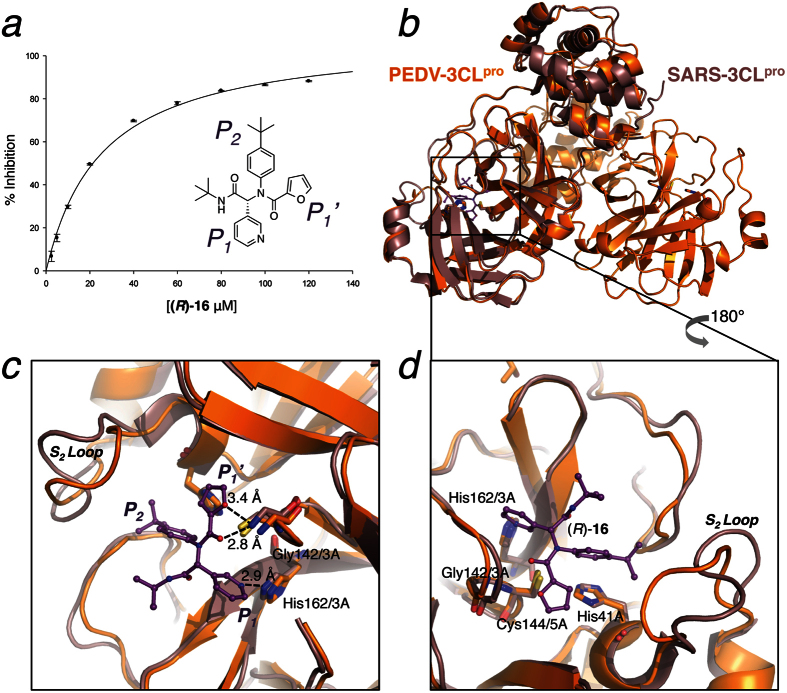
Superimposition of the X-ray structures of unbound PEDV-3CL^pro^ (colored in orange) and (*R*)-**16** bound SARS-3CL^pro^ (colored in mauve, PDB entry 3v3m). (*R*)-**16** is shown as stick representation in deep violet and colored according to atom type. (**a**) Chemical structure of (*R*)-**16** with representative IC_50_ data for (*R*)-**16** against PEDV-3CL^pro^ displayed. (**b**) Superimposition of PEDV-3CL^pro^ and SARS-3CL^pro^, where both are displayed in ribbon with the residues of the catalytic dyad shown as sticks. (**c**) (*R*)-**16** interactions with the SARS-3CL^pro^ residues where hydrogen-bonding interactions are represented as sticks and the distances between heteroatoms are labeled in angstroms (Å). (**d**) Overall active site architecture of PEDV-3CL^pro^ and SARS-3CL^pro^, where the catalytic dyad and residues involved in (*R*)-**16** binding are shown in sticks.

Our observations of the overall conserved structural features surrounding the PEDV-3CL^pro^ catalytic dyad, but subtle differences in the overall active site architecture, made us curious as to whether one of the inhibitors we developed for SARS-3CL^pro^ would also inhibit PEDV-3CL^pro^^15^. SARS-3CL^pro^ belongs to a different phylogenetic lineage than PEDV-3CL^pro^ and shares lower sequence identity (45.4%) with PEDV-3CL^pro^; however, we reasoned that the similar tertiary structure and conserved active site architecture of 3CL^pro^’s would allow for inhibition by the same molecule. We therefore tested the inhibition of PEDV-3CL^pro^ by (*R*)-**16**, which was developed as a non-covalent inhibitor against SARS-3CL^pro^ with potential broad-spectrum activity (Fig. 3a)^15^. We found (*R*)-**16** to inhibit PEDV-3CL^pro^ with an IC_50_ value of 25.4 ± 1.4 μM, where the representative curve for (*R*)-**16** inhibition of PEDV-3CL^pro^ is shown in Fig. 3a and the data for (*R*)-**16** inhibition of SARS-3CL^pro^ has been previously published^15^. The previously reported IC_50_ of (*R*)-**16** against SARS-3CL^pro^ is 1.5 ± 0.3 μM^15^, which indicates a ~17-fold weaker interaction of (*R*)-**16** with PEDV-3CL^pro^. The inhibition of PEDV-3CL^pro^ by (*R*)-**16**, though weak, is significant because it indicates that the development of non-covalent broad-spectrum inhibitors of 3CL^pro^’s may be possible.

To gain structural insights into how (*R*)-**16** may bind to PEDV-3CL^pro^, a structural alignment of the X-ray structure of SARS-3CL^pro^:(*R*)-**16** complex with PEDV-3CL^pro^ was generated (Fig. 3b). LSQ superposition of unbound PEDV-3CL^pro^ and inhibitor-bound SARS-3CL^pro^ (PDB entry 3V3M)^15^, where PEDV-3CL^pro^ is from the alpha-coronavirus phylogenetic group and SARS-3CL^pro^ is from the beta-coronavirus phylogenetic subgroup 2b, resulted in an all-atom RMSD value of 5.18 Å and a C-alpha RMSD value of 4.98 Å. The structural alignment shows that, similarly to 229E-3CL^pro^ and FIPV-3CL^pro^, the overall active site architectures of SARS-3CL^pro^ and PEDV-3CL^pro^ are largely similar despite their lower sequence identity (Fig. 3b–d), and the residues directly involved in binding to (*R*)-**16** via hydrogen-bonding interactions are all conserved (Gly142 and His162 in PEDV-3CL^pro^ and Gly143 and His163 in SARS-3CL^pro^, Fig. 3c,d).

The active sites of both SARS-3CL^pro^ and PEDV-3CL^pro^ are solvent exposed on one side with the residues of their catalytic dyads, His41 and Cys144 (for PEDV-3CL^pro^) or Cys145 (for SARS-3CL^pro^), located in almost identical structural space (Fig. 3c,d). The superposition shows that (*R*)-**16** similarly occupies the S_2_-S_1_′ subpockets in each of the 3CL^pro^ active sites, where the *tert*-butyl amide resides in the channel leading to the active site, the *tert*-butylanilido group (*P*_*2*_) sits in the S_2_ pocket, the 3-pyridyl group (*P*_*1*_) resides in the S_1_ region, and the tetrahydrofuran (*P*_1_′) occupies the S_1_′ subsite (Fig. 3c,d). As shown in the SARS-3CL^pro^ 3V3M structure, the 3-pyridyl nitrogen of (*R*)-**16** acts as a hydrogen-bond acceptor for SARS-3CL^pro^ His163, with a distance of 2.8 Å between heteroatoms (Fig. 3d). This interaction is likely conserved in the inhibition of PEDV-3CL^pro^ by (*R*)-**16** as His162 of PEDV-3CL^pro^ is in an almost identical structural location as that of SARS-3CL^pro^ His163. Additionally, the bifurcated interaction between the furan ring oxygen and the amide carbonyl oxygen of (*R*)-**16** and the backbone amide NH of SARS-3CL^pro^ Gly143 is mimicked by PEDV-3CL^pro^ Gly142 (Fig. 3d).

**Figure 4:**
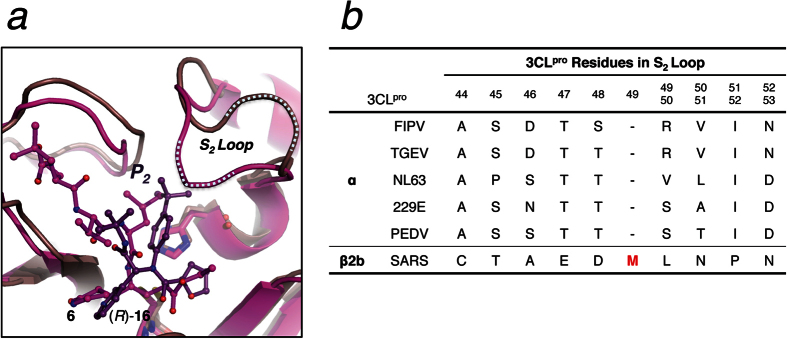
Analysis of S_2_ loops in coronaviral 3CL^pro^’s. Where SARS-3CL^pro^ is colored in mauve and FIPV-3CL^pro^ in magenta and displayed in ribbon representation (PDB entries 3v3m and 4zro, respectively). Inhibitors **6** and (*R*)-**16** are shown in sticks and colored according to atom type. (**a**) Superposition of inhibitor bound forms of FIPV- and SARS-3CL^pro^, where **6** is shown in magenta and (*R*)-**16** in deep violet. The regions of the S_2_ loop that are comprised by residues 47 and 48 in each FIPV- and SARS-3CL^pro^ are indicated by a cyan dashed line. (**b**) Analysis of S_2_ loops from various coronaviral 3CL^pro^’s.

Though (*R*)-**16** apparently binds in the same orientation in the PEDV-3CL^pro^ active site as it does in the SARS-3CL^pro^ active site, and likely utilizes the same hydrogen-bonding interactions, the IC_50_ of (*R*)-**16** against PEDV-3CL^pro^ is 17-fold higher than against SARS-3CL^pro^. We were therefore curious if the difference in position of the S_2_ loop between PEDV- and SARS-3CL^pro^ may be important for (*R*)-**16** binding. A structural alignment of the ligand-bound structures of FIPV- and SARS-3CL^pro^ (4ZRO and 3V3M, respectively) shows that compounds **6** and (*R*)-**16** bind to the respective 3CL^pro^’s by positioning their sterically bulky, hydrophobic groups in the S_2_ subsite (leucine and t-butylanilido, respectively; Fig. 4a). This suggests that hydrophobic interactions between the inhibitor or substrate and residues of the S_2_ loop are important for their binding.

We then analyzed the reported X-ray structures and sequences for other alpha-coronavirus 3CL^pro^’s in the PDB databank including the enzymes from the following coronaviruses: FIPV, transmissible gastroenteritis virus (TGEV), and human coronaviruses NL63 and 229E^12^,^13^,^14^,^15^,^16^,^17^,^18^,^19^,^20^. A sequence comparison of the S_2_ loops of FIPV-, TGEV-, NL63-, 229E-, PEDV-, and SARS-3CL^pro^ shows that the SARS-3CL^pro^ S_2_ loop is one residue longer than those from the alpha-coronaviruses and shares no sequence identity with FIPV-, TGEV-, NL63-, 229E- and PEDV-3CL^pro^ (Fig. 4b). Additionally, the S_2_ loop of SARS-3CL^pro^ contains anionic amino acids, Glu and Asp, at positions 47 and 48, respectively. These variations in the S_2_ loop between the alpha-coronavirus 3CL^pro^’s and SARS-3CL^pro^ may explain the large observed difference in the IC_50_ value of (*R*)-**16** against PEDV-3CL^pro^ as compared to SARS-3CL^pro^ (25.4 ± 1.4 μM vs. 1.5 ± 0.3 μM, respectively). Furthermore, the combination of the increase in length and charge properties of the S_2_ loop of SARS-3CL^pro^ relative to that of the alpha-coronavirus 3CL^pro^’s likely changes the size and shape of the S_2_ binding pocket and therefore may allow for increased variability at the P_2_ site of inhibitor and substrate molecules.

**Figure 5:**
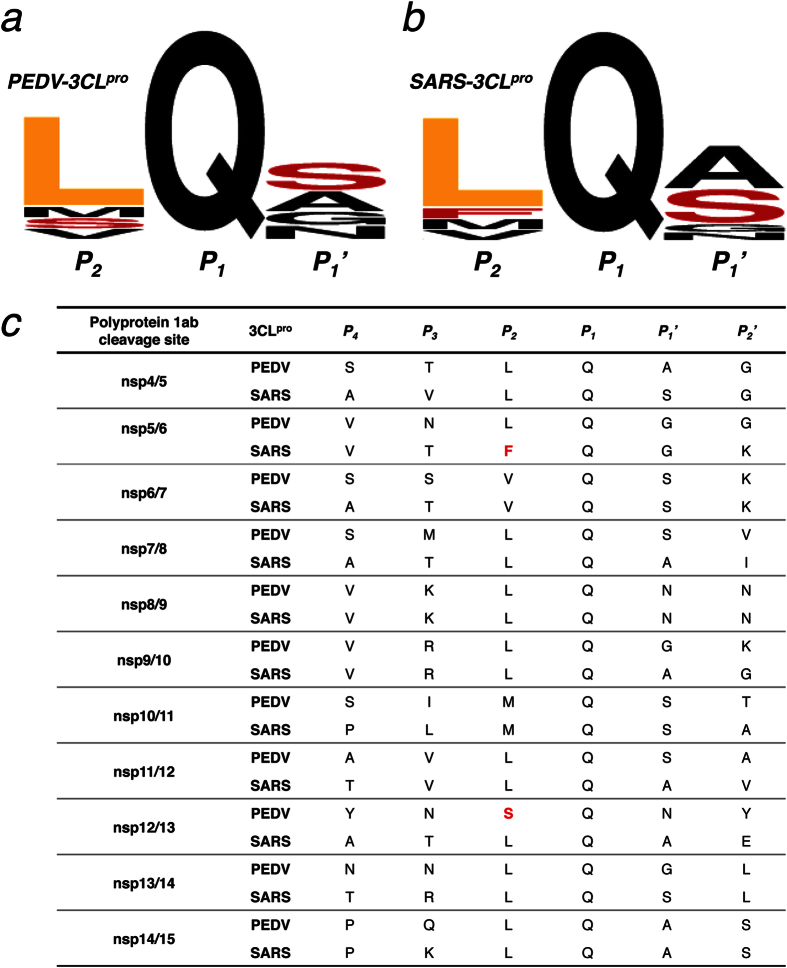
PEDV and SARS polyprotein 1ab recognition cleavage sites. (**a,b**) Sequence logos showing amino acid conservation for the eleven polyprotein 1ab cleavage sites of PEDV- and SARS-3CL^pro^, respectively, generated using the WebLogo server. (**c**) The full eleven polyprotein 1ab recognition sequences for PEDV- and SARS-3CL^pro^.

To further investigate the variation in the S_2_-subsite and relationship to inhibitor and substrate binding, we examined the recognition cleavage sequences in polyprotein 1ab of both PEDV- and SARS-3CL^pro^ (Fig. 5)^21^,^22^. The S_2_-subsite residue preference is identical for PEDV- and SARS-3CL^pro^ in most all cleavage sites, with the exception of nsp5/6 and nsp12/13. SARS-3CL^pro^ can accommodate a larger P_2_-phenylalanine, e.g. at the nsp5/6 cleavage site, while PEDV-3CL^pro^ recognizes residues only as large as a P_2_-leucine at this position in the polyprotein. At the nsp12/13 cleavage site, PEDV polyprotein 1ab has a serine at the P_2_ site, while SARS polyprotein 1ab has a P_2_-leucine. A larger size of the S_2_ pocket in SARS-3CL^pro^ is consistent with the SARS polyprotein 1ab having a phenylalanine residue at the P_2_ position of the nsp5/6 cleavage site. This larger S_2_-pocket in SARS-3CL^pro^ may allow for better binding of (*R*)-**16**, allowing the bulky *t*-butylanilido group to take full advantage of hydrophobic interactions within the S_2_ subsite and position (*R*)-**16** to optimize hydrogen-bonding with SARS-3CL^pro^ Gly143 and His163.

## Conclusions

In this study, we have determined the X-ray crystal structure of an unbound form of PEDV-3CL^pro^ to 2.10 Å resolution. We found the structure of PEDV-3CL^pro^ to be similar to the X-ray structure of the unbound human coronavirus 229E 3CL^pro^, which belongs to the same coronaviral phylogenetic subgroup as PEDV-3CL^pro^. To investigate the role of inhibitor binding on the overall architecture of the catalytic site, we generated a superimposition of PEDV-3CL^pro^ with FIPV-3CL^pro^ (PDB entry 4zro), which belongs to the same alpha-coronavirus phylogenetic group as PEDV-3CL^pro^ and shares 61.9% sequence identity, in complex with the covalent inhibitor **6**. We observed little difference between the active site architectures of unbound PEDV-3CL^pro^ and the inhibitor-bound form of FIPV-3CL^pro^, except for differences in the position and composition of a loop comprising the S_2_ subsite, which was also observed in comparison to the 229E-3CL^pro^ structure.

We tested PEDV-3CL^pro^ for inhibition by a known SARS-3CL^pro^ inhibitor, compound (*R*)-**16** and found (*R*)-**16** to be capable of inhibiting PEDV-3CL^pro^ although the IC_50_ value was roughly 17-fold higher than the reported IC_50_ for (*R*)-**16** against SARS-3CL^pro^. Structural comparison of SARS-3CL^pro^ bound with (*R*)-**16** and PEDV-3CL^pro^ in its unbound form revealed that the residues that directly interact with (*R*)-**16** in SARS-3CL^pro^ are conserved in PEDV-3CL^pro^. Additional analysis of the S_2_ loop across alpha-coronavirus 3CL^pro^’s and SARS-3CL^pro^ proved that the sequence identity of the S_2_ loop is not conserved across alpha- and beta-coronaviral 3CL^pro^’s and the SARS-3CL^pro^ S_2_ loop is one residue longer than that of the alpha-coronaviral 3CL^pro^’s, which likely increases the effective volume of the SARS-3CL^pro^ S_2_ protease subsite relative to the alpha-coronaviral 3CL^pro^ S_2_ subsites. These findings provide a potential explanation for the roughly 17-fold weaker inhibition of PEDV-3CL^pro^ by (*R*)-**16** compared to SARS-3CL^pro^.

This work advances our understanding of the subtle, but important, structural differences between 3C-like proteases from different coronaviral phylogenetic groups and contributes to the broader structural knowledge of coronaviral 3CL^pro^’s. Small structural changes have been shown to be essential in enzymatic catalysis and an understanding of these such differences is also very pertinent for the design of both broad-spectrum and selective coronaviral 3CL^pro^ inhibitors for the treatment of coronaviral infection^23^.

## Methods

### Protein expression and purification

The gene encoding the 3CL^pro^ of PEDV (residues 2998–3299 in the Porcine epidemic diarrhea virus polyprotein AHA38151.1)^24^ was codon optimized for expression *E. coli* and cloned into pET-11a expression vector with an N-terminal (His)_6_-tag followed by nsp4-/5 auto-cleavage site by BioBasic Inc. This pET-11a PEDV-3CL^pro^ construct was used because it results in the expression of PEDV-3CL^pro^ without an N-terminal or C-terminal extension.

E*. coli* BL21(DE3) cells were transformed with the pET-11a PEDV-3CL^pro^ plasmid and then grown at 25 °C for 24 hours in 500 mL Super LB media (3 g potassium phosphate monobasic, 6 g sodium phosphate dibasic, 20 g tryptone, 5 g yeast extracts, 5 g sodium in 1 L water, pH 7.2 adjusted with 1 M NaOH) that was also supplemented with 1 mL 100 mg mL^−1^ carbenicillin, 25 mL 8% lactose, 10 mL 60% glycerol, and 5 mL of 10% glucose per 1 L of expression culture. The cells were harvested by centrifugation (8,400 *g* for 20 min) to yield 14.5 g L^−1^ of cells. The cell pellet was then re-suspended in Buffer A (50 mM Tris pH 7.5, 0.2 M ammonium sulfate, 0.05 mM EDTA, 5 mM BME) containing 1 mg mL^−1^ lysozyme, where 5 mL of Buffer A was used per 1 g cell pellet. After the cells were homogenized, they were lysed via sonication for a total of 10 minutes with 10 s pulses and 20 s delays at 50% amplitude using a Branson Digital Sonifier.

The cell lysate was clarified by pelleting the cell debris via centrifugation (28,960 *g*, 4 °C, 20 minutes).

The resultant supernatant was loaded onto a 60 mL Phenyl Sepharose 6 Fast Flow HiSub (GE Healthcare) column equilibrated with Buffer A. Protein was eluted with a linear gradient to 100% Buffer B (20 mM Tris pH 7.5, 0.05 mM EDTA, 5 mM BME) over five column volumes (300 mL) collecting 5 mL fractions. Fractions containing PEDV-3CL^pro^ enzymatic activity were pooled and loaded onto a 60 mL DEAE Sepharose Fast Flow (GE Healthcare) column equilibrated with Buffer B. Protein was eluted with a linear gradient to 50% Buffer C (50 mM Tris pH 7.5, 1 M sodium chloride, 0.05 mM EDTA, 5 mM BME, 10% glycerol) over five column volumes (300 mL) collecting 5 mL fractions. Fractions containing pure PEDV-3CL^pro^, as judged by SDS-PAGE and specific activity, were pooled, dialyzed into storage buffer (25 mM HEPES, pH 7.5, 2.5 mM DTT, 10% glycerol), and concentrated using a spin concentrator (Millipore) with a 10 kDa molecular weight cutoff membrane. The protein was flash-frozen in liquid nitrogen and then stored at −80 °C in a freezer until further use (Fig. 1a). Prior to crystallization, PEDV-3CL^pro^ was concentrated to 4.0 mg mL^−1^ using a spin concentrator (10 kDa cutoff membrane, Millipore) and loaded onto a 24 mL Superdex 75 (GE Healthcare) size exclusion chromatography column equilibrated with 25 mM HEPES pH 7.5 and 2.5 mM DTT. Peak fractions containing PEDV-3CL^pro^, as determined via visualization of SDS-PAGE and confirmed by protein activity assay, were pooled and concentrated to protein concentrations of 1.5, 2.8, and 4.0 mg mL^−1^ for crystallization.

### Crystallization

The PEGS II Suite (Qiagen) sparse-matrix screen was used to screen for initial PEDV-3CL^pro^ crystallization conditions. Crystallization trials (150 nL protein and 150 nL of crystallization solution) were set up in a 96-well sitting drop tray (Intelli-Plate 96) using a TTP LabTech Mosquito® liquid robotics system at PEDV-3CL^pro^ concentrations of 1.5, 2.8, and 4.0 mg mL^−1^ in buffer containing 25 mM HEPES pH 7.5 and 2.5 mM DTT. An initial hit of clusters of needle-like crystals was obtained at 20 °C from the PEGS II Suite condition No. 50 consisting of 10% (w/v) PEG-4000 and 20% (w/v) isopropanol. Crystals were also obtained at each of the other PEDV-3CL^pro^ concentrations. Optimization of this condition was achieved by varying the concentration of both isopropanol and PEG-4000 between 15–30% and 7–22% respectively, where diffraction quality crystals grew at a variety of conditions from this round of optimization. The best crystals grew at 20 °C in 25% isopropanol and 22% PEG-4000 at a protein concentration of 1.5 mg mL^−1^ and appeared after 24–72 hours (Fig. 4). Crystals were transferred using 0.05–0.1 μm nylon loops to small drops containing the crystallization solution plus the cryo-protectant, which was 15% 2-methyl-2,4-pentanediol (MPD). After cryo-protection, the crystals were remounted into the nylon loop and rapidly flash-cooled in liquid nitrogen.

### Data collection and structure refinement

X-ray diffraction data were collected on LS-CAT beamline 21-ID-F at the Advanced Photon Source (APS) at Argonne National Laboratory, Argonne, Illinois, USA. Data were indexed, integrated and scaled using *HKL-*2000^25^ and the resulting structure factor amplitudes were used for molecular replacement (MR). The program Phaser-MR (simple interface) module of *PHENIX*^26^ was used to perform MR and the X-ray structure of the human coronavirus 229E-3CL^pro^ in complex with the peptidomimetic compound EPDTC (PDB entry 2zu2^13^) with ligands and waters removed was used as a search model. Iterative rounds of manual building and structural refinement were completed using *PHENIX*, and manual inspection, rebuilding and the addition of water molecules were accomplished using the programs *Coot*^27^ and the refinement module of *PHENIX*. The final data collection and refinement statistics are summarized in Table 1.

### Inhibitor characterization

The compound (*R*)-**16** was synthesized according to Jacob *et al*.^15^. Inhibition of PEDV-3CL^pro^ by (*R*)-**16** at a concentration of 100 μM was first tested in an enzymatic assay containing the following buffer (50 mM HEPES, 0.1 mg/mL BSA, 0.01% TritonX-100, 1 mM DTT). The assays were carried out in triplicate using Costar 3694 EIA/RIA 96-Well half-area, flat bottom, black polystyrene plates from Corning Incorporated. 1 μL of 100X inhibitor stock in DMSO was added to 79 μL of enzyme in assay buffer and the enzyme-inhibitor mixture was incubated for 10 minutes. The reaction was initiated by the addition of 20 μL of 10 μM UIVT3 substrate, a custom synthesized Förster resonance energy transfer peptide substrate with the following sequence: HilyteFluor^TM^488-ESARLQSGLRKAK-QXL520^TM^-NH_2_, producing final concentrations of 100 nM for the 3CL^pro^ enzyme and 2 μM UIVT3 substrate. The increase in fluorescence intensity of the reaction was then measured over a period of 10 minutes as relative fluorescence units (RFU_t_). An excitation wavelength of 485 (bandwidth of 20 nm) and an emission wavelength at 528 (bandwidth of 20 nm) was used to monitor the reactions using a BioTek Synergy H1 multimode microplate reader. The percent inhibition of the PEDV-3CL^pro^ was determined using the following equation (1):


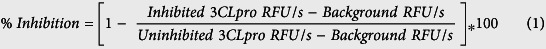


The IC_50_ value of (*R*)-**16** against PEDV-3CL^pro^ was determined at ambient temperature using 100 μL assays conducted in the following buffer: 50 mM HEPES, 0.1 mg/mL BSA, 0.01% TritonX-100, 1 mM DTT. Kinetic assays were carried out in triplicate and in Costar 3694 EIA/RIA 96-Well Half Area, flat bottom, black polystyrene plates from Corning Incorporated. (*R*)-**16** was tested at concentrations of 0.313, 0.652, 1.25, 2.5, 5.0, 10, 20, 40, 60, 80, 100 and 120 μM by adding 1 μL of 100X inhibitor stock in DMSO to 79 μL of enzyme in assay buffer and then incubating the enzyme-inhibitor mixture for 10 minutes. The reaction was initiated by the addition of 20 μL of 10 μM UIVT3 substrate, producing final concentrations of 100 nM for 3CL^pro^ and 2 μM for the UIVT3 substrate. The increase in fluorescence intensity over time during the reaction was then measured.

The average percent inhibition of PEDV-3CL^pro^ was then calculated from triplicate data, and the final averaged data with standard deviation were then plotted as a function of inhibitor concentration. The data were fit to the following equation (2) to determine the IC_50_:


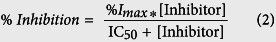


where %I_*max*_ is the percent maximum inhibition of 3CL^pro^. The errors in the IC_50_ and %I_*max*_ values are the errors in the fitted parameters resulting from fitting of the equations to the data.

## Additional Information

**Accession codes:** Model coordinates and structure factor amplitudes for the X-ray crystal structure of unbound PEDV-3CLpro were deposited in the Protein Data Bank (acc. #: 5HYO).

**How to cite this article**: St. John, S. E. *et al*. X-Ray Structure and Inhibition of 3C-like Protease from Porcine Epidemic Diarrhea Virus. *Sci. Rep.* **6**, 25961; doi: 10.1038/srep25961 (2016).

## Figures and Tables

**Table 1:** Data-collection and refinement statistics for PEDV-3CL^pro^.

**PDB entry**	**5HYO (unbound PEDV-3CL**^**pro**^)
Data-collection parameters	
No. of crystals	1
Temperature (K)	100
Beamline	21-ID-F, APS
Detector	MAR Mosaic 300
Wavelength (Å)	0.9878
Resolution limit (Å)	2.10
Space group	P2_1_2_1_2_1_
*a, b, c* (Å)	58.57, 76.29, 119.17
Data-processing statistics	
Data resolution range (Å)	150–2.10 (2.14–2.10)
Total reflections collected	687,631
Unique reflections	31,793
% Completeness	94.4 (93.3)
*R*_*merge*_ (%)	8.2 (83.8)
*R*_*pim*_(%)	3.4 (35.0)
Average *I/σ(I)*	26.2 (1.9)
CC1/2	74.0
CC*	92.2
*Refinement statistics*	
Data resolution range (Å)	46.5–2.10 (2.46–2.10)
Reflections in working set	29,146
Reflections in test set	1,947
*R*_*work*_ (%)	20.4
*R*_*free*_ (%)	26.9
Ramachandran plot most favored (%)	92.8
Ramachandran plot allowed (%)	5.9
Ramachandran plot outliers (%)	1.3
No. protein molecules in model	2
No. of H_2_O molecules	183
Wilson *B* factor (Å^2^)	34.2

Values in parentheses are for the last shell.
